# Supplementary figures and images for: Glial immune-related pathways mediate effects of closed head traumatic brain injury on behavior and lethality in Drosophila
Source: PLoS Biol. 2022 Jan 26;20(1):e3001456. doi: 10.1371/journal.pbio.3001456 (PMC8791498; doi:10.1371/journal.pbio.3001456)

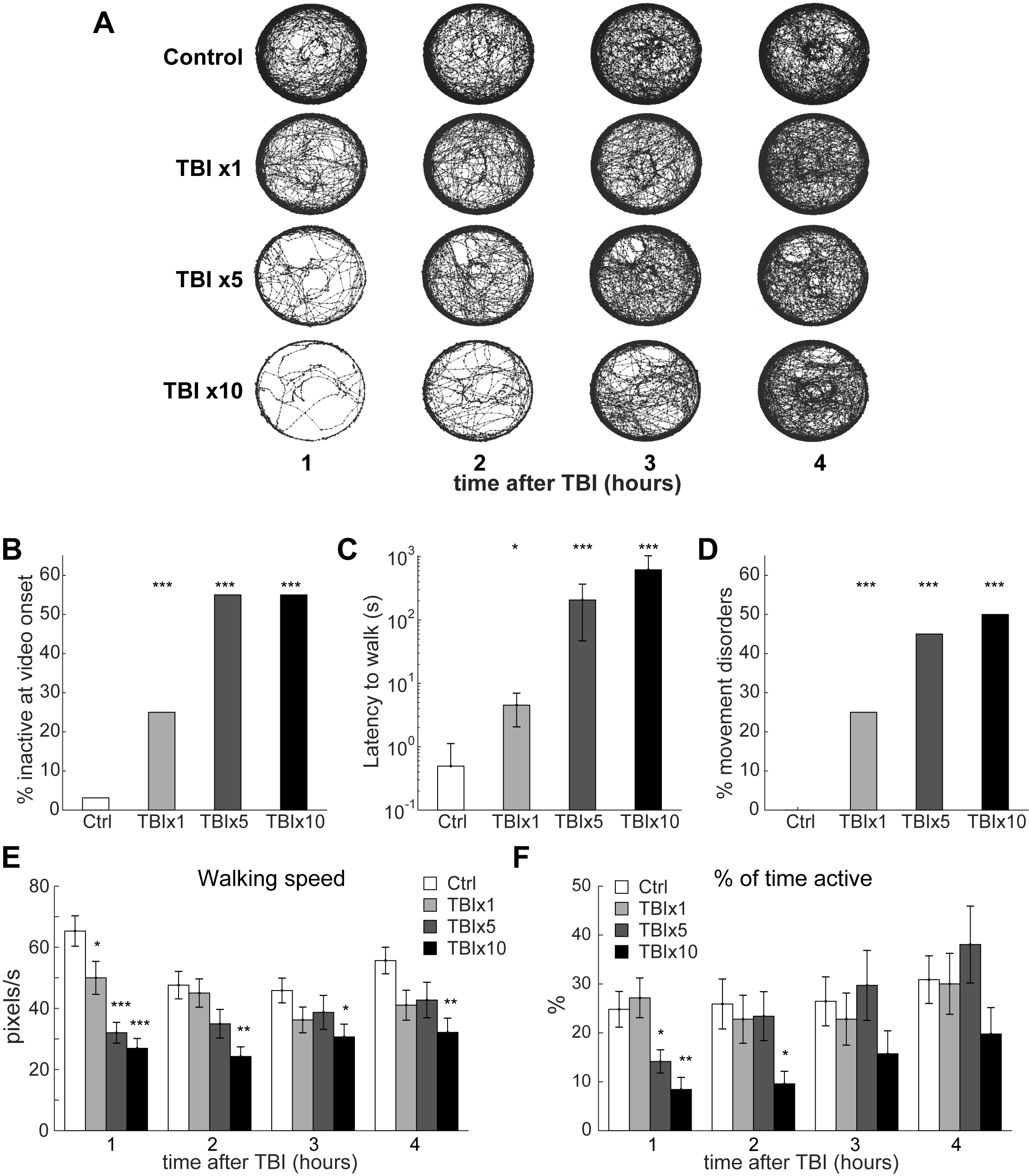

Supplement: S1 Fig — (A) Representative position traces for single flies during the first 4 hours immediately after TBI onset, for controls as well as flies in the TBIx1, TBIx5, and TBIx10 conditions. (B) TBI resulted in a dose-dependent number of flies being immobile immediately after TBI onset (*** p < 0.001 chi-squared test). (C) It took these flies second (TBIx1) to minutes (TBIx5 and TBIx10) to become active (* p < 0.05, *** p < 0.001, t test). (D) Locomotor defects (circling, slow walking, sideways walking, backwards walking, and jumping) occurred shortly after TBI onset, in a dose-dependent manner. Locomotor defects only were only observed in flies that were immobile after TBI (*** p < 0.001 chi-squared test). (E) Walking speed was reduced in all 3 groups during the first hour post-TBI, but the TBIx1 and TBIx5 groups had recovered by the second hour. Walking speed remained impaired for all 4 hours in the TBIx10 group. (F) Overall activity (% of time active) was significantly reduced in the TBIx5 and TBIx10 groups for the first hour after TBI, but unaffected in the TBIx1 group (* p < 0.05, ** p < 0.01, *** p < 0.001, t test). n = 20–24 per TBI group, 32 controls. Error bars indicate SEM. Movie extracted data can be found in S1 Data. TBI, traumatic brain injury. (TIF) [file pbio.3001456.s001.tif]

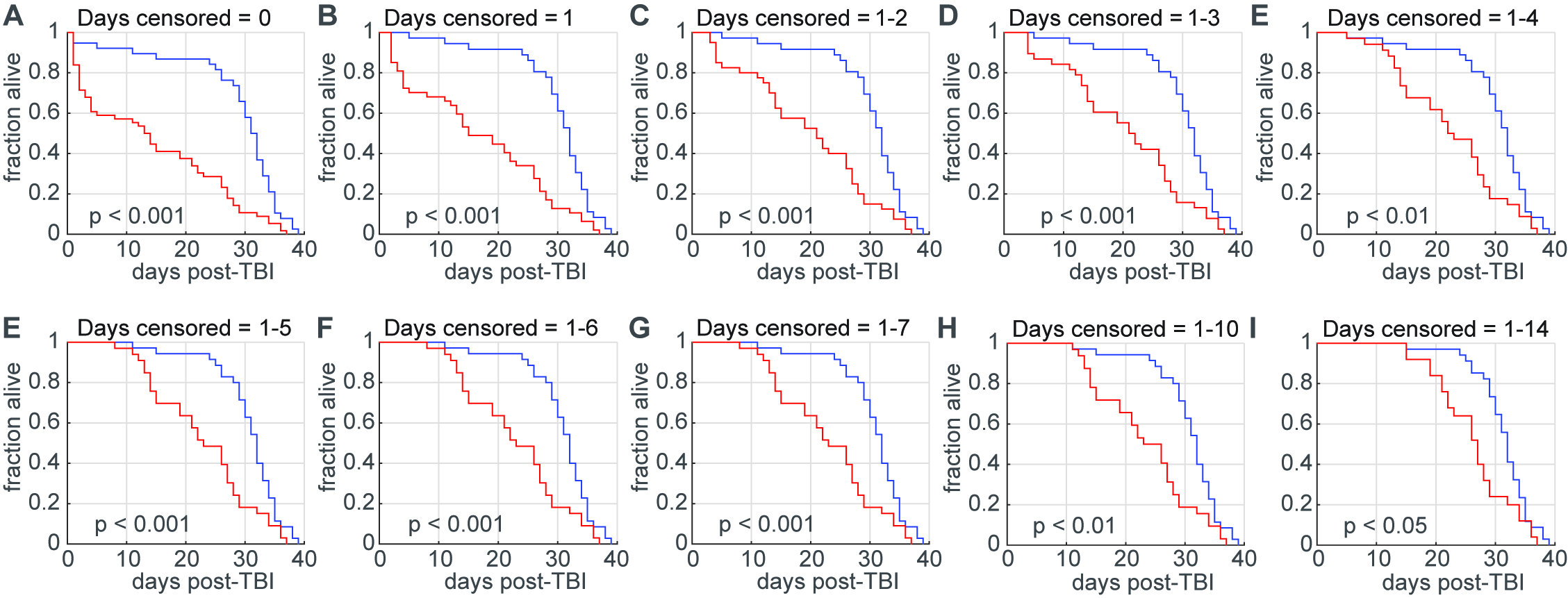

Supplement: S2 Fig — To test whether increased mortality due to TBI can be explained by early deaths, we set mortality to zero cumulatively for the first 2 weeks post-TBI. In all instances, we see significantly increased mortality in the TBI-treated group (log-rank test), indicating that the observed increase in mortality is not due to early deaths only. All figure-related data are located in S2 Data. TBI, traumatic brain injury. (TIF) [file pbio.3001456.s002.tif]

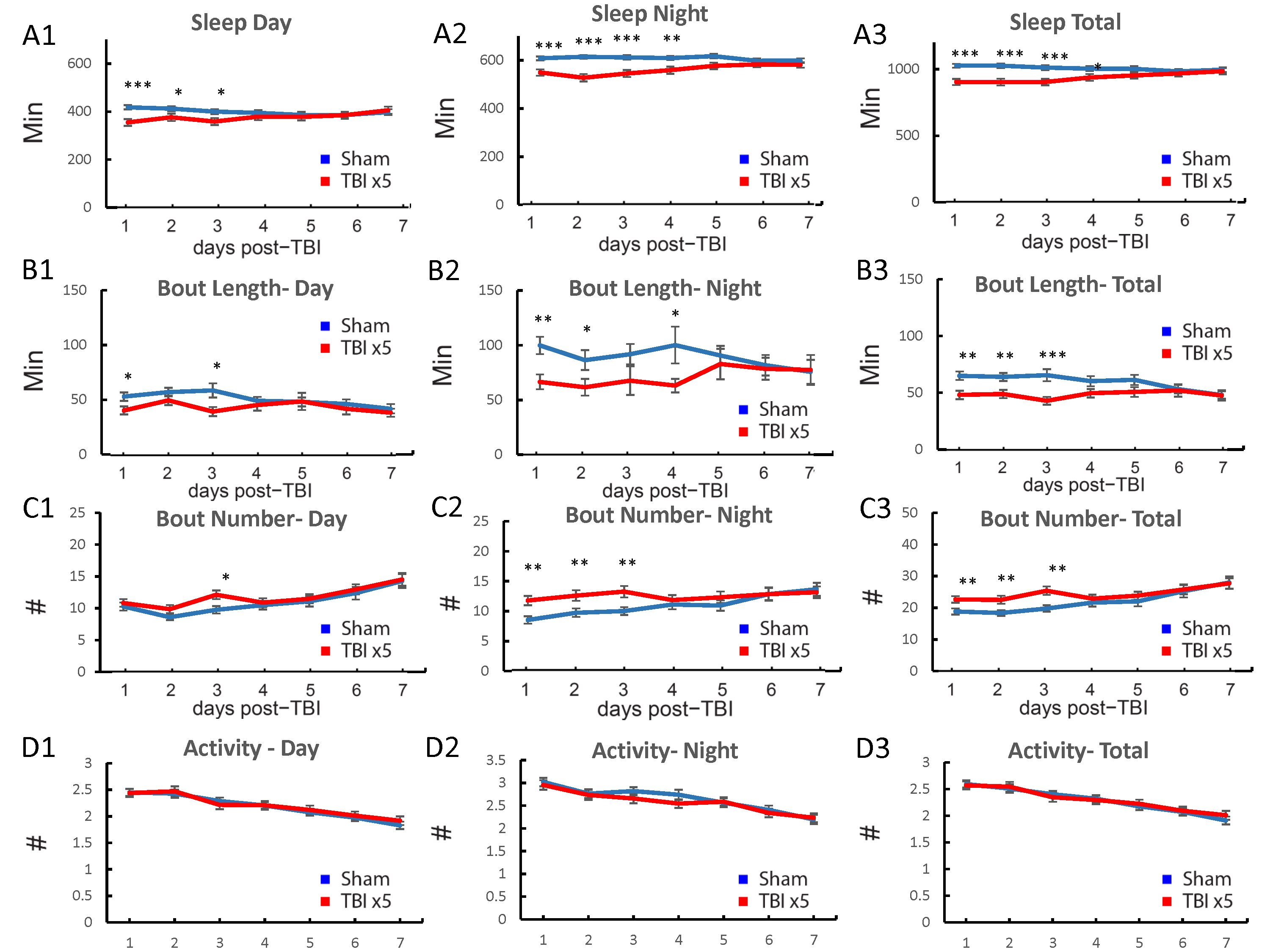

Supplement: S3 Fig — To test whether sleep affect flies that survive our 7-day sleep experiment differently than flies that die during this experiment, we split our sleep data in survivors and dying flies. Sleep data for survivors is shown here. (A1–3) Total sleep during the day, night and total sleep shows that post-TBI is mostly decreased during the night, for up to 4 days post TBI. (B1–3) Average sleep bout length was modestly reduced, and (C1–3) sleep bout numbers were increased, suggesting that sleep is both decreased and fragmented for the first 3 days after TBI. (D1–3) Wake activity was not affected by TBI during the first 7 days post-TBI. n = 67 sham-treated and 56 TBI flies. *** p < 0.001, ** p < 0.01 by t tests with Bonferroni correction. Error bars indicate SEM. All figure-related data are located in S3 Data. TBI, traumatic brain injury. (TIF) [file pbio.3001456.s003.tif]

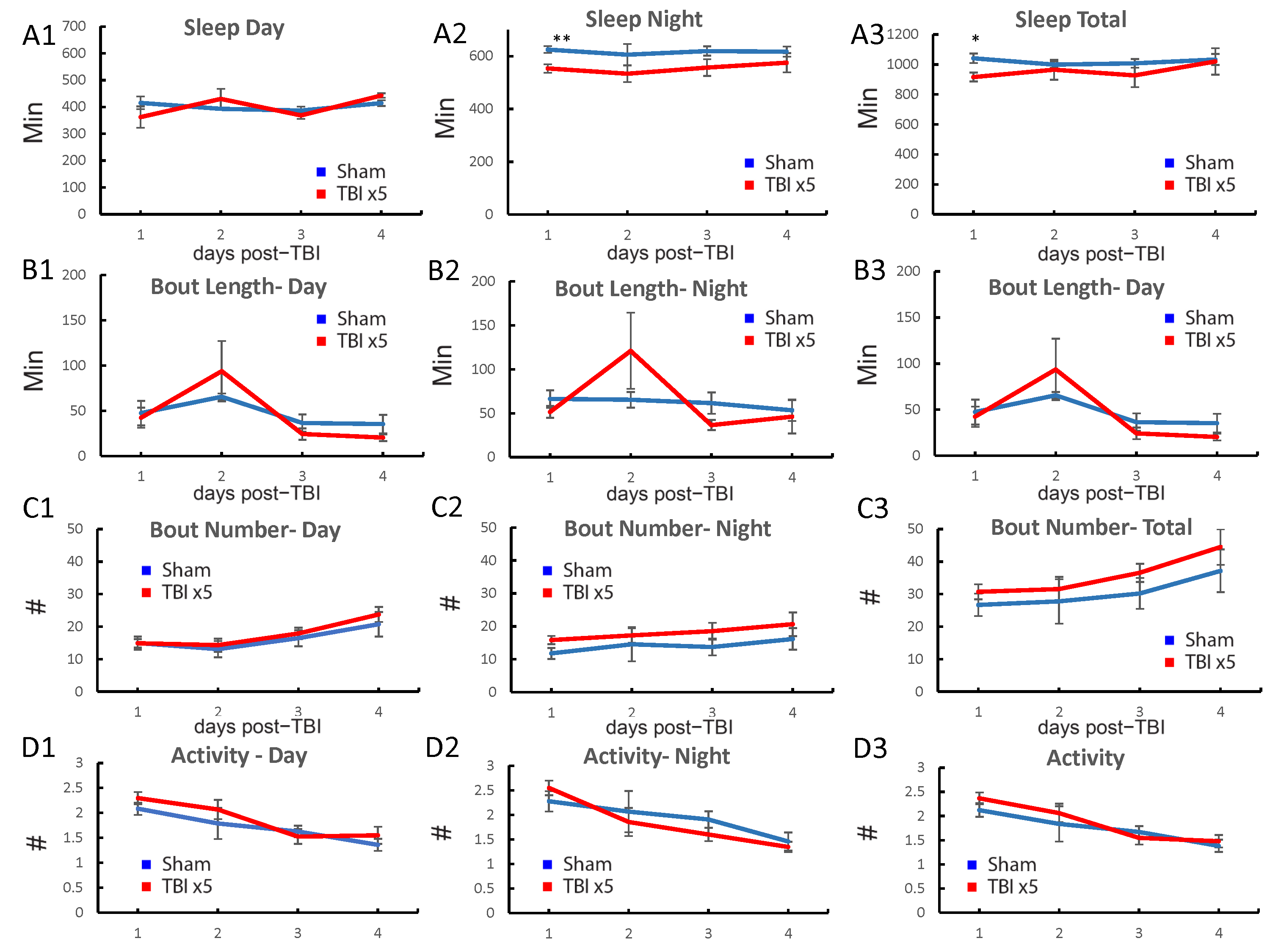

Supplement: S4 Fig — To test whether sleep affect flies that survive our 7-day sleep experiment differently than flies that die during this experiment, we split our sleep data in survivors and dying flies. Sleep data for dying flies is shown here. (A1–3) Total sleep during the day, night and total sleep shows that post-TBI is increased during the day on days 2 and 3 post-TBI. (B1–3) Average sleep bout length was strongly increased during the day, but (C1–3) sleep bout numbers were unaffected, suggesting that sleep more consolidated during post-TBI days 2 and 3. (D1–3) Wake activity during the day was not affected by TBI during the first 3 days post-TBI, indicating that the observed sleep effect is not due to decreased locomotion. n = 17 sham-treated and 40 TBI flies. *** p < 0.001, ** p < 0.01 by t tests with Bonferroni correction. Error bars indicate SEM. All figure-related data are located in S3 Data. TBI, traumatic brain injury. (TIF) [file pbio.3001456.s004.tif]

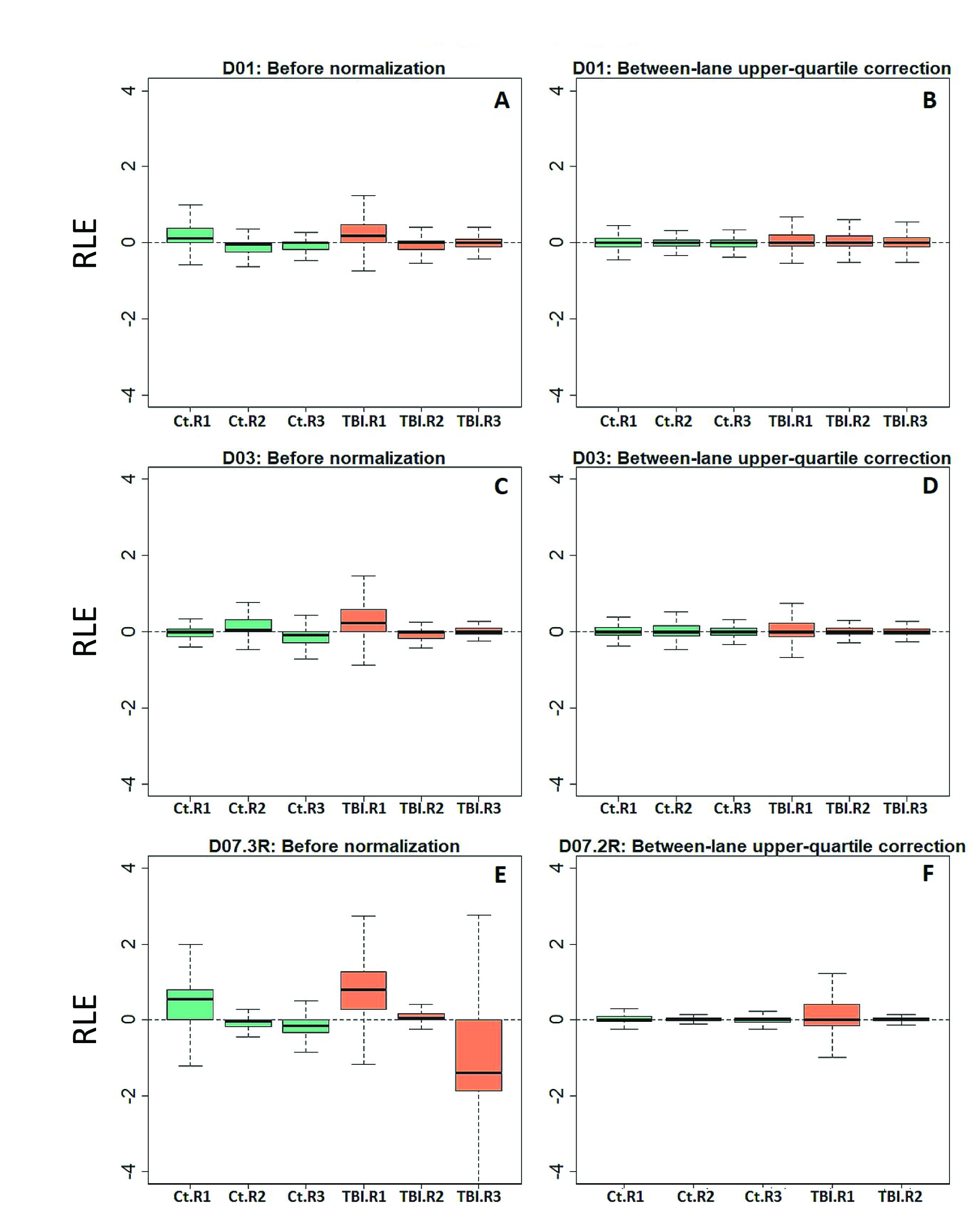

Supplement: S5 Fig — RLE plot of raw and normalized glial expression data. Control (green) and TBI (orange) biological replicates for days 1, 3, and 7 post-TBI. Correction was performed using the UQ normalization method. Due to the high variability in TBI replicate 3 on day 7, this replicate was discarded. RNA-seq data were deposited under accession number GSE164377. RLE, relative log expression; TBI, traumatic brain injury; UQ, upper-quartile. (TIF) [file pbio.3001456.s005.tif]

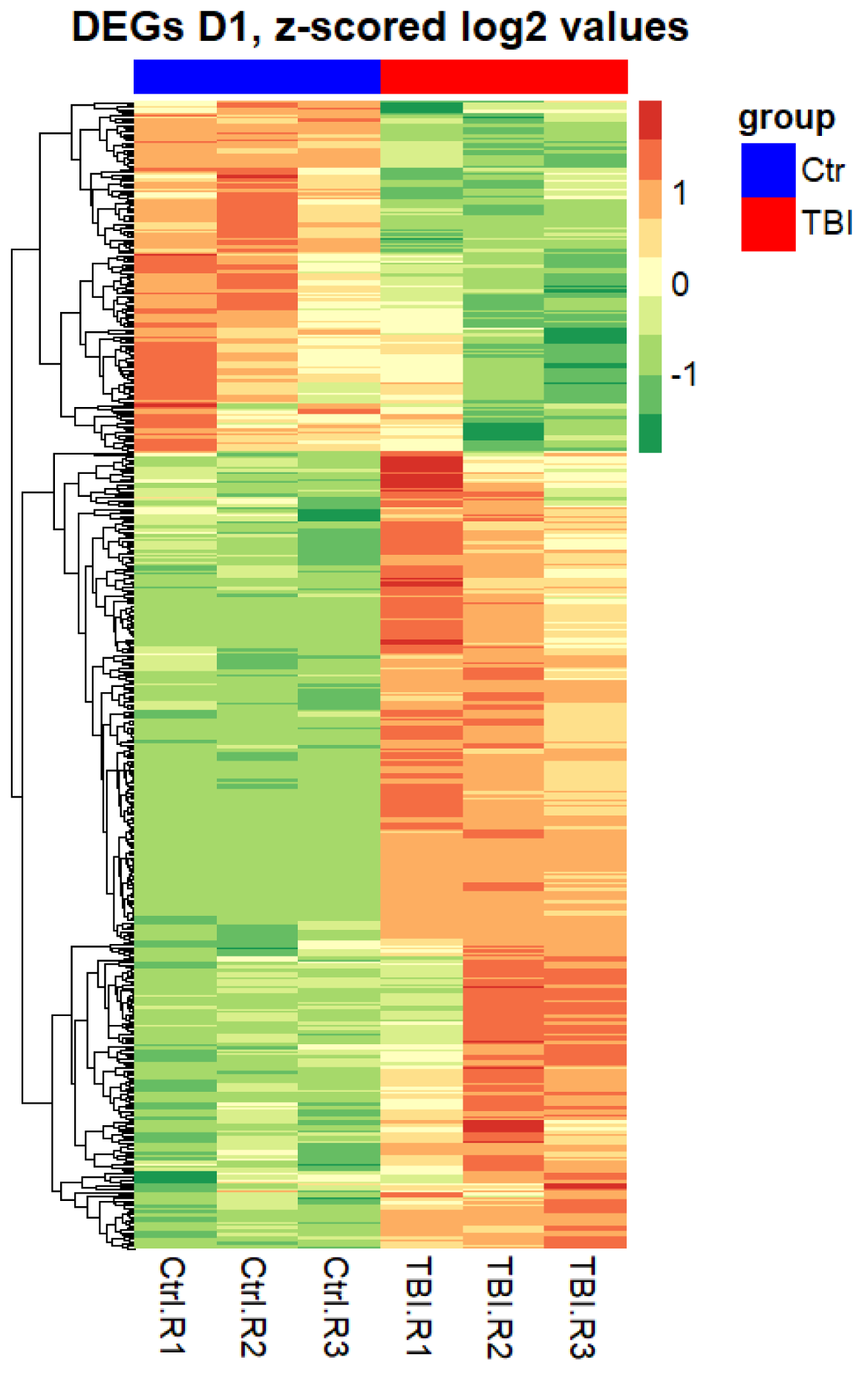

Supplement: S6 Fig — Panels present clustering of DEGs for day 1 post-TBI. Gene expression level presented as z-scored log2(X+1) transformed values; control replicates in blue, TBI replicates in red. All figure-related data are located in S4 Data. DEG, differentially expressed gene; TBI, traumatic brain injury. (TIF) [file pbio.3001456.s006.tif]

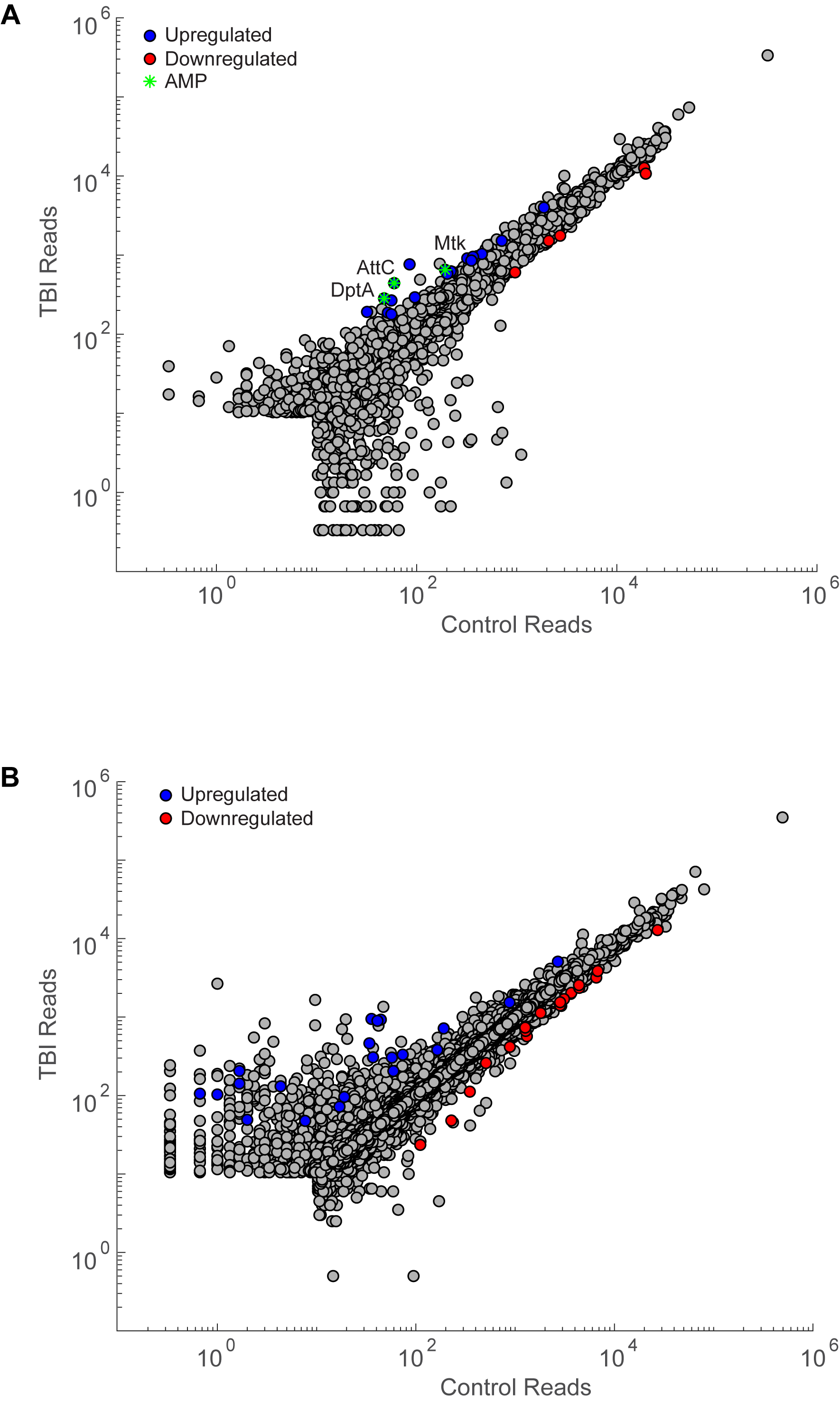

Supplement: S7 Fig — Scatter plot for glial genes where average reads in the control condition are plotted against average reads 3 days (A) or 7 days (B) after TBI (blue dots, log₂fold change ≥ 0.6, Benjamini-adjusted p < 0.1) or down-regulated (red dots, log₂fold change ≤ −0.6, Benjamini-adjusted p < 0.1) 24 hours after TBI induction. AMPs are indicated with green asterisks. Genes with average reads <10 in both control and TBI condition were excluded. All figure-related data are located in S4 Data. AMP, antimicrobial peptide; TBI, traumatic brain injury. (TIF) [file pbio.3001456.s007.tif]

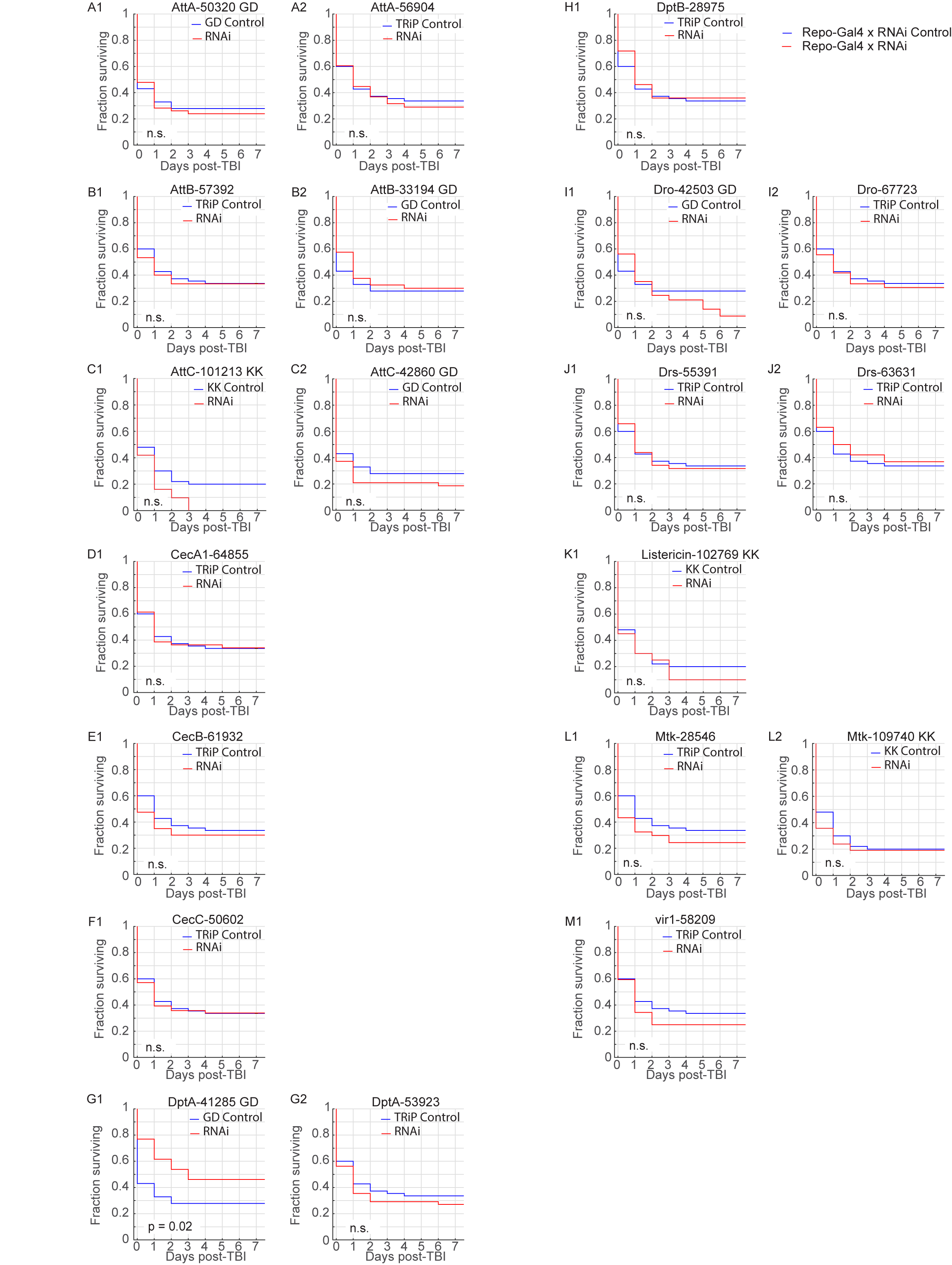

Supplement: S8 Fig — Kaplan–Meier plots for glia-specific RNAi-mediated knockdown of AMPs. Repo>RNAi lines are compared to Repo>RNAi control lines using a log-rank test. n.s. = not significant. (A) Attacin-A, (B) Attacin-B, (C) Attacin-C, (D) Cecropin-A, (E) Cecropin-B, (F) Cecropin-C, (G) Diptericin-A, (H) Diptericin-B, (I) Drosocin, (J) Drosomycin, (K) Listericin, (L) Metchnikowin, (M) virus-induced RNA 1. All figure-related data are located in S7 Data. AMP, antimicrobial peptide; TBI, traumatic brain injury. (TIF) [file pbio.3001456.s008.tif]

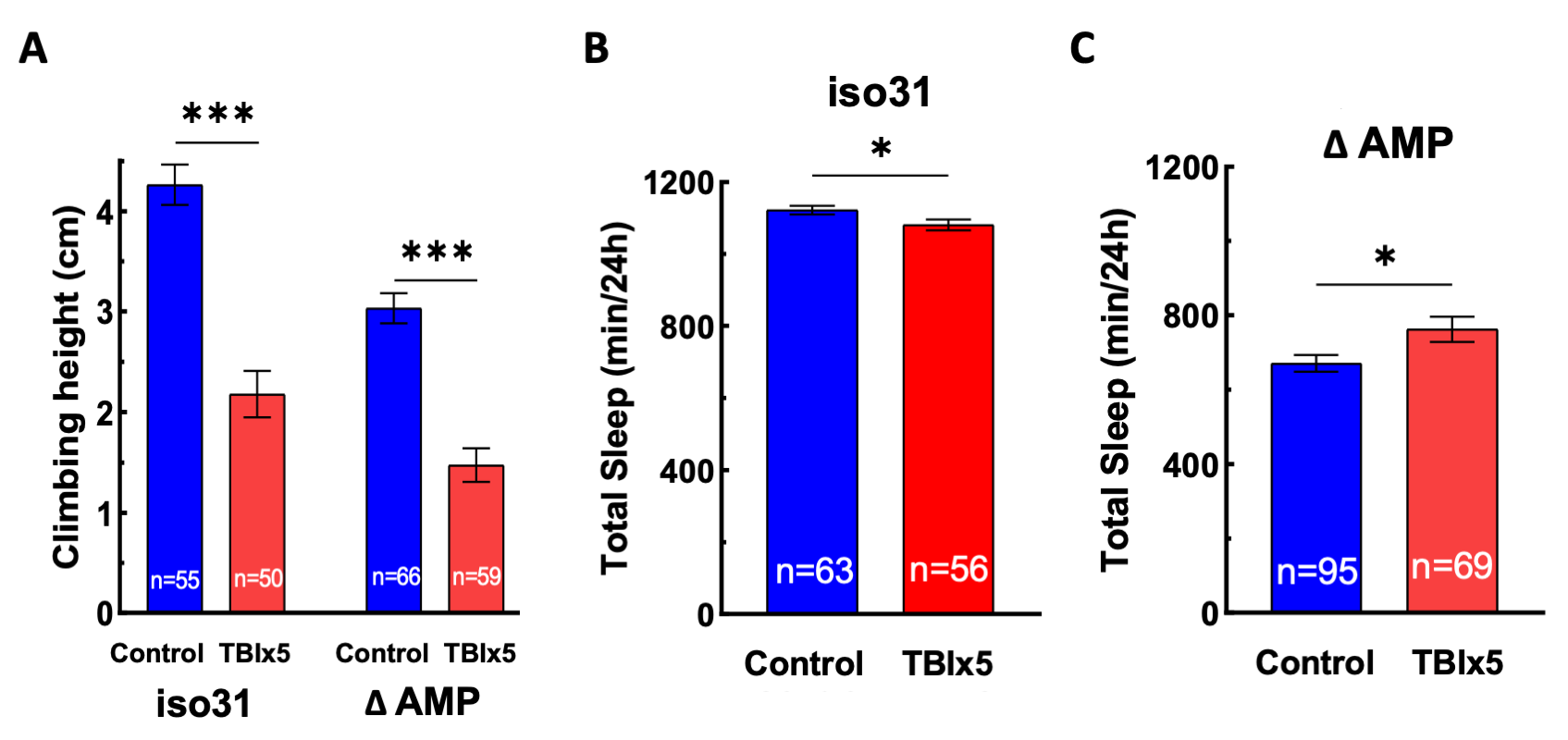

Supplement: S9 Fig — (A) ΔAMP null mutants show decreased climbing 24 hours after TBI, similar to controls (n = 50–66) (B) Sleep is decreased in controls 24 hour after TBI (C) but is increased in ΔAMP null mutants (n = 56–95). *** p < 0.001, * p < 0.05 by t tests with Bonferroni correction. Error bars indicate SEM. All figure-related data are located in S2 Data. AMP, antimicrobial peptide; TBI, traumatic brain injury. (TIF) [file pbio.3001456.s009.tif]
