# Supplementary material for: Glial immune-related pathways mediate effects of closed head traumatic brain injury on behavior and lethality in Drosophila
Source: PLoS Biol. 2022 Jan 26;20(1):e3001456. doi: 10.1371/journal.pbio.3001456 (PMC8791498; doi:10.1371/journal.pbio.3001456)
Supplement: Attachment — Submitted filename: Reviewer response BvA2RA3.pdf [file pbio.3001456.s023.pdf]

Reviewers' comments in *italics* and our response in plain purple text. Changes in the main text are also indicated in purple.

Rev. 1:

The authors describe a method to deliver strikes to the head of anesthetized flies to establish a novel traumatic brain injury (TBI) model. They performed a number of tests to determine the action of TBI on survival, negative geotaxis, sleep and gene expression. Sequence analysis identified an upregulation of a larger number of genes including those involved in the innate immune response which decreases after 7 days post TBI. Only one gene is persistently downregulated on all days tested (CG40470). The authors then tested the role of NF-kB signaling and performed TBI in a Relish null mutant. This showed an increased immediate mortality and moreover indicated that Post-TBI behavioral phenotypes are NF-kB - dependent. In addition, they showed that AMP deficiency has an impact on the TBI survival.

TBI is an important subject to study and work in Drosophila might potentially help to understand human pathology. However, I do have some concerns regarding the brain specific trauma induction, this approach in my view is likely to generate disruptions of (1) nerves running in the neck of the fly and disruptions in the foregut which is also located in the neck. In addition, the forces used in this study (8.34 N) appear quite high in particular when comparing to the forces obtained in the device of Katzenberger et al., (2013, 2 N). This should at least be discussed. And more importantly it must be analyzed whether the neck is not damaged.

We now acknowledge in the Discussion the potential that other head tissues including the neck may have been damaged in our assay as is the case also for mammalian TBI assays and other assays used to address TBI in flies. We believe that most if not all of the phenotypes observed can be attributed to brain damage (i.e. we observe cell death in the brain and glial gene expression changes) but acknowledge that we cannot exclude damage to other tissues/structures contributing.

We also make note in the Discussion of the difference in force used in our assay relative to the Katzenberger paper. We may need a higher force because brain damage is caused by the direct impact of the solenoid to the fly head, where the fly head moves with the solenoid (sup movie 1) rather than full body injury or compression injuries used in the other Drosophila TBI assays.

We also note in the Discussion that our assay allows us to deliver strikes directly to fly head, without having to anesthetize flies, thus avoiding anesthesia effects on behavior and TBI outcomes.

The present work provides a number of interesting finding - which however are not entirely new. The Bonini group just published a paper "Dynamic neural and glial responses of a head-specific model for traumatic brain injury in Drosophila" presenting a head-specific TBI model (Saikumar et al., PNAS 2020). In addition, Wassarman and colleagues published paper in 2015 where they showed that TBI results in blood-brain barrier permeability defects (Katzenberger 2015). Both of these two important papers are not mentioned at all - despite the fact that 130 references are presented.

We have added references to both studies throughout the revised manuscript. The Bonini paper was published just prior to our submission. We thank the reviewer for pointing out this issue.

The finding that genes of innate immunity response are upregulated has been made before. The authors should compare their sequence data with the previously published datasets. The specificity of the TRAP-seq method should be documented (e.g. expression of alrm, wunen-2, repo, gliotactin and moody should be compared to neuronal gene expression e.g. nsyb or elav). It should be stated that gliotactin is not a marker for peripheral glial cells but is expressed by the subperineurial glia - as moody and wunen-2 is not a marker for astrocytes but shows a rather broad expression in the adult brain (see Stein Aerts data single cell seq dataset). The finding that CG40470 is the only gene that is persistently downregulated in all days tested is interesting but unfortunately not analyzed in further detail.

Thank you for these remarks. We have added a comparison to previous datasets with regards to upregulated immune response genes in the Discussion section.

With regards to TRAP-seq, we have updated Fig 4A in the following ways: we have removed wunen and moody as markers for glial expression and we have added elav and nsyb as neuronal markers (expression levels for both were not significantly altered after TBI). *

We agree that CG40470 is an interesting gene to follow up on in future studies and make note of this finding in the text.

Rev. 2:

An extensive literature links TBI to defects in behavior, neural function, and health. However, TBI-induced cellular responses and functional consequences remain an area of intense interest. To this end, van Alphen et al. have developed an effective TBI assay in Drosophila which recapitulates many canonical consequences of TBI, including neuronal death and locomotor impairment. Promisingly, this TBI model is sufficiently robust to elicit significant changes in gene expression in glia, thereby allowing the authors to identify genetic candidates for manipulation. Indeed, disrupting different classes of anti-microbial peptides (AMPs) either improved or impaired resilience to TBI, potentially reconciling previous conflicting studies. Broadly, the authors have designed and validated a novel, precise assay that opens the possibility of screening for TBI response mechanisms and to understand TBI recovery.

Major comments:
1. One complicated feature of TBI is the heterogeneity of outcomes in injured animals, which is also demonstrated in this study. Here, it appears that a large proportion of flies die within ~1-2 days post-TBI, after which it seems that the remaining flies have only a slightly accelerated aging curve. It is appropriate, then, to separate these flies into different groups for analysis. For example, do flies that die rapidly exhibit stronger sleep impairments? If these rapidly-dying flies are separated from the analysis, does the survival curve of the remaining TBI-treated flies look more comparable to sham-treated controls?

This is an excellent point. We reanalyzed our mortality and sleep data to test whether flies that die early behave differently in these assays.

For the mortality data, we removed early deaths from both controls and TBI flies cumulatively, for up to two weeks after TBI, and performed log rank test on the remaining flies. In all cases, survival rate is still significantly decreased in the TBI group, suggesting that the increased mortality is not entirely due to flies that die early. This data is now in Sup Fig 2.

For sleep data, we removed all flies that died during the seven days from the analysis and analyzed sleep for two conditions:

Condition 1 compares sleep in flies that survived TBI to controls. Here we see that the reduced sleep phenotype is still present validating our original finding.

Condition 2 compares sleep in all flies that died during the seven days post TBI to controls. Here we observe an interesting phenotype. While sleep is unaffected at day 1 post-TBI, sleep is significantly increased during post-TBI days 2 and 3. This is only due to increased sleep during the light phase, where bout lengths are significantly increased. Night time sleep is unaffected. Also, wake activity during the light phase does not differ between controls and TBI-treated flies, indicating that the increased sleep phenotype is not due to impaired locomotion. Thus the flies that die exhibit a distinct sleep phenotype from those that do not die.

These data are now in Sup Figs 3 and 4.

2. Figure 2C shows a significant elevation in TUNEL staining to label apoptotic cells in the brain after TBI. Because the authors focus their sequencing studies on glia, it would be informative to test whether the

TUNEL-positive cells are neuronal or glial. Do glia activate AMP expression in response to neuronal death? Or to their own apoptosis?

This is an interesting point and we note the ambiguity of the identity of the cells in the text. Experimentally addressing this issue would be beyond the scope of this study.

3. The authors have demonstrated in Figure 6 that the mortality and behavioral phenotypes can be partially dissociated. However, this is not consistently addressed. The AMP mutants used in Figure 7 are only tested for mortality; it remains unclear if these AMP mutants, either individually or in combination, could also account for the behavioral phenotypes. Do flies in the "ABC" class from Figure 7 show changes in climbing and sleep behavior after TBI?

We tested whether TBI affects climbing and sleep in the ΔAMP flies. Baseline climbing did not differ between ΔAMP flies and controls without TBI and both groups showed a similar reduction in climbing behavior after TBI. However, TBI affected sleep in the opposite direction in ΔAMP flies. Where the wild-type controls show a reduction in sleep 24 hours after TBI, ΔAMP flies show increased sleep post-TBI. Thus, AMPs appear to be important for mediating TBI effects on sleep. This data is now in Sup Fig 8.

4. The authors use TUNEL staining for validation of the TBI model but do not address whether the immune genes that affect survival after TBI also affect neuronal death. To provide a more complete characterization of the role of immune genes, the authors could compare neuronal death in relish mutants and wild-type flies after TBI.

This is also an interesting point and one that is worthwhile for a follow up study (noted in the Discussion) but beyond the scope of this study.

5. The authors claim that the immune response mediates survival after TBI. However, it is possible that immune responses are not playing an active role after TBI but rather that the mutants are more sensitized/susceptible to TBI. The authors should either discuss this caveat in the text or address this experimentally using inducible genetic systems to knock down relish starting ~24h before TBI.

Yes, we cannot rule out a role for immune mutants rendering the flies susceptible to TBI. However, the finding that immune gene expression changes after TBI suggests a role in mediating the response. We more fully discuss this in the text.

6. Sleep architecture data (Figs. 3D-F) should show daytime and night time results separately.

Sleep architecture data are now split by daytime and nighttime. Results are described in response to reviewer 1.

Minor comments:
1. Are flies that die over the course of the 7-day sleep experiment excluded from the entire dataset, or only from the days after they died? These flies might provide insight into whether the severity of behavioral changes might correlate with mortality.

Flies that die over the course of the 7-day sleep experiment were included in the dataset. As noted above, (Major Comment #1) we found that excluding these flies from the dataset did not change our conclusions. However, analyzing sleep only in the flies that died over the course of the experiment showed that sleep is increased in this group on days 2 and 3 post-TBI.

2. The authors argue that TBI induces an immune response. However, it has also been demonstrated that sleep disturbances themselves can induce the upregulation of immune genes (eg - Dissel et al. 2015; Williams et al. 2007). Therefore, it is possible that TBI might indirectly increase immune responses. by

disturbing sleep. This may merit discussion in the text.

This is a really interesting point, we have added some discussion of the possibility that TBI-induced immune responses may be secondary to their effects on sleep. We have added a paragraph to the discussion on sleep responses after injury.

3. Figure 7 lacks n values

We have added n values to this figure.

Dissel S, Seugnet L, Thimgan MS, Silverman N, Angadi V, et al. 2015. Differential activation of immune factors in neurons and glia contribute to individual differences in resilience/vulnerability to sleep disruption. Brain Behav Immun. 47:75-85
Williams JA, Sathyanarayanan S, Hendricks JC, Sehgal A. 2007. Interaction between sleep and the immune response in Drosophila: a role for the NFkappaB relish. Sleep. 30(4):389-400

Rev. 3:
In this manuscript, Van Alphen et al describe a novel model for Traumatic Brain Injury (TBI) and characterize the molecular signatures associated with the injury. While flies have been used as a model to study TBI for some time, the approach described here represents a significant advance over current methodologies because the force and location of the injury can be precisely controlled in an awake animal. The symptoms described, including loss of coordination, shortened life span, and disrupted sleep phenoocpy those in mammals. The manuscript applies cell-type specific RNAseq/TRAPseq to define the molecular signatures of TBI within glial cells and validate a number of genes involved in immune response as contributing to the effects on mortality or function. Overall the manuscript is well written and technically sound. This manuscript will be of broad interest to researchers interested in neurodegeneration, sleep and fly genetics and provides a solid foundation for futurestudies on TBI. In my opinion, the impact of the manuscript in its current form is sufficient to justify publication. However, the experimental characterization of the assay, and some of the functional validation of TBI-regulated genes could be strengthened. I have included suggestions below, though some may be beyond the scope of this manuscript.

Major comments:
1) Given the novelty of this assay the authors might consider additional descriptions of the behaviors themselves. For example, quantifying differences in behavior immediately following the TBI events through the first few hours of recovery.

This is an excellent point. To address it, we induced TBI at three intensities (x1, x5, x10) and used video tracking in individual flies, housed in small petri dishes, to record locomotor behavior in the four hours immediately after TBI induction. Locomotion metrics were compared to sham treated controls.

We found that after TBI, ~25% flies in the TBIx1 condition are immobile, versus ~55% in the TBIx5 and x10 conditions. Flies in the x1 condition started moving within seconds, while flies in the x5 and x10 conditions started moving after minutes (3.3 and 10 min respectively). Walking speed was reduced in all three groups during the first hour post-TBI, but the TBIx1 and x5 groups had recovered by the second hour. Walking speed remained impaired for all four hours in the TBIx10 group. Overall activity (% of time active) was significantly reduced in the TBIx5 and x10 groups for the first hour after TBI, but unaffected in the TBIx1 group. We also observed some locomotor defects (circling, slow walking, sideways walking, backwards walking, jumping) shortly after TBI onset, in a dose dependent manner (25%, 45% and 50% in the TBIx1, x5 and x10 groups respectively). These movement disorders only occurred in flies that were immobile immediately after TBI and were not observed in flies that immediately started walking.

This data in now shown in Sup Fig 1.

2) It is interesting that the behavioral deficits return to normal after a few days. I would be very interested to know if they have memory deficits that persist beyond this point. This may be beyond the scope of the paper, but would be worth discussing.

In their recently published Drosophila TBI model, Saikumar et al (2020) demonstrated that memory deficits, measured by courtship conditioning, are present ten days after severe TBI. Surprisingly, baseline courtship behavior was unaffected. We added a brief section to the discussion on this issue.

3) I understand the advantages of targeting the head, even with this precision is it possible to differentiate between neural injury and general stress? Is it possible that targeting a different body region would also lead to climbing/sleep deficits?

This is an interesting point which we acknowledge explicitly in the text.

4) Genetic background is certainly an important factor for sleep and longevity/aging and is therefore likely very important TBI response. Please describe efforts to account for genetic background.

We agree that genetic background is very important in sleep and, likely, the response to TBI. We have taken the following measures:

All TBI experiments were carried out in young adult (3-7 days old) male iso31 flies, an isogenic w^{1118} control strain commonly used for sleep research. Our NFkB null mutant, Rel[E20] is in a w^{1118} background, and we used w^{1118} iso31 as control. For the AMP null mutants, we compared survival rates to the iso31 control line provided by the LeMaitre lab.

We have clarified our methods section.

5) Localizing genes to subpopulations of glia would increase the impact of the findings. would be very helpful to sort TRAP-seq data based on the glial subtype that they express in. I understand this is not entirely straight forward and these data sets don't exist for all glia, but they do for Repo and Alrm, and this alone might be useful. An alternative would be to knock genes down in subsets of glia.

Testing the effect of knocking down hits from our glial TRAP-seq, both in all glia and in subpopulations is a follow up experiment we are considering but beyond the scope of the current study. We added some discussion of this in the text.

Minor Comments

1) Line 40: Is TBI really one of the leading causes of death? This seems unlikely. Also, (though perhaps too detailed to address here) I imagine most TBI deaths are in elderly patients, which leads me to wonder if the effects of TBI would differ in aged flies.

According to data compiled by the CDC, TBI is a leading cause of death among children and young adults rather than among the elderly. https://www.cdc.gov/traumaticbraininjury/pubs/tbi_report_to_congress.html

To avoid confusion, we have rephrased 'leading' to 'major' on line 40.

2) While not critical to the scientific content, Figure 1 could be improved to depict the assay. For example, a cartoon diagraming the components of the assay would be more useful to the image in A, which could be placed in the supplemental figures.

We have added a new schematic figure as suggested.

3) Line 258 describes the immediate response of flies to the TBI, and their recovery. Supplemental videos are provided but it would be very useful to quantify this given the novelty of the assay. In addition, the term 'dazed' may inadvertently imply changes in cognitive perception.

This overlaps with Major Comment 1.

We have updated the text to: “flies are often able to stand but only barely respond to tactile stimuli”

4) Figure 3. While not essential, it would be a useful control to show climbing and sleep data in animals given a single strike. Five strikes results in some death, and therefore phenotypes may derive from generalized deficiencies (although the finding that sleep returns to normal after 7 days suggests the effects are specific).

Climbing data for TBIx1 is shown in Fig 2B. There was no effect on climbing 24 hours after TBIx1. We also did not observe sleep effects after TBI x1 (data not shown).

However, our video analysis of locomotion effects in the first four hours immediately after TBI, we see that ~25% of flies are immobile immediately after TBI and flies show decreased walking velocity for the first hour post TBI. However, all locomotor effects in TBIx1 flies are gone by the 2nd hour after TBI.

5) Figure 2. When do flies die within the 24hrs following TBI? Is it immediate, or hours after? There are also some caveats about using negative geotaxis to infer sensory-motor function (though these are likely shared in rotarod studies). It does not rule out things like general arousal, endurance, or motivation.

After reanalyzing our TBI sleep data for mortality, we found that 16% of flies die immediately after, and 22% of flies die within 24 hours after TBI induction.

We acknowledge the caveats with the negative geotaxis assay.

6) Recent work from the Donlea group showed that antennal axotomy results in increased sleep. It is worth commenting on the difference in sleep phenotypes that result from each type of neural injury. This is an excellent point. Both groups demonstrated that antennal axotomy increases sleep, a process that facilitates clearance of debris, while our work demonstrates a reduction in sleep with TBI, suggesting that Wallerian degeneration is not a predominant component of TBI. We did note a transient increase in sleep in flies that die suggesting it may play a role there.

7) How was the strength of the TBI-inducing stimulus chosen?

When designing our TBI paradigm, we tested several commercially available solenoids for their ability to induce TBI and used the one that gave the best results. We may need a higher force because brain damage is caused by the direct impact of the solenoid to the fly head, where the fly head moves with the solenoid (sup movie 1) rather than full body injury or compression injuries used in the other Drosophila TBI assays.

8) In some cases the language could be more precise. E.g. line 422 'Also, quite a few members of the turanadot…'
We have fixed these issues.

Rev. 4:
In this manuscript ("Glial immune-related pathways as mediators of closed head TBI effects on behavior and lethality in Drosophila"), van Alphen and Stewart et al. describe an in vivo traumatic brain injury (TBI) model in adult fruit flies that triggers a number of quantifiable pathological and behavioral phenotypes associated with nervous system decline. The authors have performed a large scale unbiased transcriptional screen, utilizing Translating Ribosome Affinity Purification and Sequencing (TRAP-Seq) to identify genes upregulated in glial cells at several time points post-TBI. In follow-up validation experiments, they show that specific genes (NF-kB transcription factor relish and classes of secreted AMP immune factors) differentially influence TBI-induced phenotypes. Overall, this work offers an interesting new model and, most importantly, a novel data set (TRAP screen) for the glial/injury scientific community to explore and potentially define additional glial-specific signaling pathways that are invoked after closed head injury (both beneficial and detrimental pathways). However, there are some questions and issues related to analysis and methods that must be addressed before the article would be appropriate for publication.

Specific Comments for Authors:

1. The primary strength of this manuscript lies in the TRAP screen. Thus, the authors must provide a complete/comprehensive data set of the genes altered in the TBI assay. In addition, this list of differentially expressed genes with altered expression levels, p values, etc. will provide a complete picture of the efficacy of the TRAP approach. This manuscript does show that known adult glial-specific genes are upregulated in TBI animals, but, as presented, it is not clear if neuron-specific genes are also induced (and/or detected in uninjured animals due to technical/specificity challenges).

Thank you for this comment. We have added the complete data sets of genes with altered expression levels for days 1,3 and 7 post TBI as Supplementary Files 1-3. We now also show in Fig 4A that expression levels of neuro-specific gene elav is not significantly changed after TBI., and nsyb is downregulated. Other neuronal genes (bruchpilot, neurexin, neuroligin) are detected but not up- or downregulated.

2. The authors state that differential gene expression analysis was identified using a p value of 0.1 (and Log2 value of 0.6) as a threshold. This value is high. The authors should clarify why this is an appropriate cutoff value (or choose to make the cutoff more stringent, for example p value of 0.05 or below).

Thank you for your remark. We use FDR corrected p-values (adjusted p-values) to control for the false discovery rate. The significance threshold is an arbitrary value that signals how many false positives one agrees to accept. These thresholds should take into consideration the experimental data and requirements for the downstream analysis, like GO. Depending on the application and further use of the data, values between 0.01- 0.1 are generally accepted in the field [2-7]. Our choice of more relaxed FDR threshold takes into consideration correlation and range of values for replicates for analyzed conditions, as well as the fact that gene expression differences in the brain might be subtle on transcriptional level. We decided to go with the default threshold implemented in DESeq2 (FDR ≤0.1 [1]), we added additional filter in the form of the fold change ratio factor, requiring at least 50% of change (up or down) in the expression level in relation to the value in control samples, to support the biological relevance of results. These thresholds combined serve as a good indicator of observed effect, and are accepted in the field [i.e. 3, 6,7]

1) http://bioconductor.org/packages/release/bioc/vignettes/DESeq2/inst/doc/DESeq2.html
2) Jansen, R., Penninx, B., Madar, V. et al. (2016). Gene expression in major depressive disorder. Mol Psychiatry 21, 339–347.
3) Francesconi, M., & Lehner, B. (2014). The effects of genetic variation on gene expression dynamics during development. Nature, 505(7482), 208-211
4) Wingo, A. P., & Gibson, G. (2015). Blood gene expression profiles suggest altered immune function associated with symptoms of generalized anxiety disorder. Brain, behavior, and immunity, 43, 184-191.
5) Pletikos, M., Sousa, A. M., Sedmak, G., Meyer, K. A., Zhu, Y., Cheng, F., ... & Šestan, N. (2014). Temporal specification and bilaterality of human neocortical topographic gene expression. Neuron, 81(2), 321-332
6) McCabe, M., Waters, S., Morris, D., Kenny, D., Lynn, D., & Creevey, C. (2012). RNA-seq analysis of differential gene expression in liver from lactating dairy cows divergent in negative energy balance. BMC genomics, 13(1), 193
7) Tomás, A., Fernandes, L. T., Sánchez, A., & Segalés, J. (2010). Time course differential gene expression in response to porcine circovirus type 2 subclinical infection. Veterinary research, 41(1), 1-16.

3. It is still unclear to this reviewer how significance of 7 day RNAseq data samples were generated with an n value of 2. There are trend analysis statistical approaches that can be utilized (which would, for

example, track increased gene expression at days 1, 3, and 7 after injury), but it's not clear in the Methods if this type of approach was utilized.

We did not utilize trend analysis in this study. Significance of differentially expressed genes was assessed by DESeq2. We understand that two replicates per condition is not ideal and some differentially expressed genes will not be detected. Two replicates per condition in the RNA-seq experiments are accepted in the field (i.e. 8-10), and all current-age differential gene expression packages/algorithms (i.e. DESeq2[1], edgeR[11]) have no issues with calculating dispersion and assessing statistical significance using two replicates (please see vignettes and user manuals). In our TRAP RNA-seq experiment we use three replicates per condition, which is above the minimal requirement for the algorithms analyzing differential gene expression and accepted in the field (i.e. 12-14). We disclosed in the manuscript that only one of the three replicates for Day7 did not pass quality assessment, leaving us with two usable replicates, which do meet minimal requirements of DESeq2 for assessing the significance.

8) Comoglio F, Schlumpf T, Schmid V, Rohs R, Beisel C, Paro R (2015) High-Resolution Profiling of Drosophila Replication Start Sites Reveals a DNA Shape and Chromatin Signature of Metazoan Origins. Cell Reports, 11(5), 821-834
9) Pankova K, Borst A (2016) RNA-Seq Transcriptome Analysis of Direction-Selective T4/T5 Neurons in Drosophila. PLOS ONE 11(9): e0163986. https://doi.org/10.1371/journal.pone.0163986
10) Castillo, J.C., Creasy, T., Kumari, P. et al. Drosophila anti-nematode and antibacterial immune regulators revealed by RNA-Seq. BMC Genomics 16, 519 (2015).
11) https://www.bioconductor.org/packages/release/bioc/vignettes/edgeR/inst/doc/edgeRUsersGuide.pdf
12) Zhu, L., Liao, S. E., Ai, Y., & Fukunaga, R. (2019). RNA methyltransferase BCDIN3D is crucial for female fertility and miRNA and mRNA profiles in Drosophila ovaries. PloS one, 14(5), e0217603.
13) Liao, S. E., Ai, Y., & Fukunaga, R. (2018). An RNA-binding protein Blanks plays important roles in defining small RNA and mRNA profiles in Drosophila testes. Heliyon, 4(7), e00706.
14) Tindell, S.J., Rouchka, E.C. & Arkov, A.L. Glial granules contain germline proteins in the Drosophila brain, which regulate brain transcriptome. Commun Biol 3, 699 (2020)

4. Fast QC data from Illumini Seq runs should be included in the Results

We have uploaded our Fast QC data to the GEO repository (Series record GSE164377). It will be publicly available on 5/31.

5. The authors should include a discussion of the recent publication (Saikumar et al., PNAS, July 2020), which describes a similar TBI protocol and neurodegeneration analysis in adult Drosophila.

We have added discussion of this to our revised manuscript.

6. In Figure 2D, are these TUNEL-positive values in the entire central brain - or optical sections?

These are TUNEL-positive values for the entire central brain. We have added this to the relevant method section.

7. This manuscript aims to explore the role of glia in TBI-induced changes in physiology. This model will be substantially strengthened if the authors can perform a subset of experiments that specifically alter the expression of genes identified in the TRAP screen (for example, components of the NF-kB pathway) and show they (at least partially) recapitulate the phenotypes of whole animal mutants. It is not surprising that this would be difficult to perform with the AMP factors due to functional redundancy.

Yes, we did attempt this for the AMPs and did not identify any significant effects of knocking down individual AMPs. As mentioned by the reviewer functional redundancy is a likely factor here. We have added these data as new Sup Fig 8.

8. The authors provide a range of behavioral readouts for TBI in control versus NF-kB mutants. It would be informative to complement these results with a physiological analysis. For example, how is cell death (TUNEL) affected? Are there changes in synaptic connectivity (e.g. nc82 staining), which often precede cell death in neurodegeneration models.

These follow up mechanistic studies will certainly be of interest for a future study and we discuss these as future experiments in our discussion but are beyond the scope of our current work.

9. Minor point: Authors should describe head homogenization, step pre-bead incubation, and RT details (cDNA synthesis) in greater detail.

We have added the following to the TRAP-seq method section:

Heads were homogenized for 3 minutes by Pellet Pestle™ Cordless Motor.

The cDNA was used as template for T7 transcriptase to amplify the original RNA. We synthesized 1st and 2nd strand cDNA from RNA first with Superscript III and DNA polymerase. Then we amplified the RNA by synthesizing more RNA from the cDNA template with T7 RNA polymerase. Amplified RNA was purified with RNeasy Mini Kit (QIAGEN). A detailed procedure for amplification can be in found in [49]. After the second round of cDNA synthesis from amplified RNA, the cDNA was submitted to HGAC at the University of Chicago for library preparation and sequencing.
