# Supplementary material for: Glial immune-related pathways mediate effects of closed head traumatic brain injury on behavior and lethality in Drosophila
Source: PLoS Biol. 2022 Jan 26;20(1):e3001456. doi: 10.1371/journal.pbio.3001456 (PMC8791498; doi:10.1371/journal.pbio.3001456)
Supplement: Attachment — Submitted filename: Response to Reviewers_PLOS Biology_82721.docx [file pbio.3001456.s024.docx]

In response to the editorial comments and the PLoS Data Policy, we have

1. Updated the title (see above),
2. Provided supplemental data files or indicated the database where data are freely available.
3. Added data locations in the figure legends.
4. Indicated that the first author is currently deceased.
5. We noted some minor issues in the sleep analysis that, when updated, do not change the overall results in Fig. 3, S3 as well as Fig. 6. In Fig. S4 the flies dying during the run do not exhibit strong sleep effects after TBI compared to their surviving counterparts which do. Updated versions of the figures have been uploaded.
6. In addition, there was a minor error in the Venn diagram in Figure 4. An updated figure has been uploaded.
7. In the case of Figure S9 we were not able to find the underlying data for panels S9A and S9B. After discussion with the editor, we have repeated all S9 experiments. These experiments replicate the major findings included in submission R2: (S9A) climbing is decreased after 5X TBI in both wild-type and AMP deletion mutants, (S9B) sleep is decreased in iso31 control one day after 5X TBI, and (S9C) sleep is increased in AMP deletion mutants one day after 5X TBI.
8. We have also added Bridget Lear, Shiju Sisobhan, and Zuoheng Qin as co-authors given their roles in data analysis and/or repeating AMP deletion sleep and climbing assays (Figure S9).
